# Supplementary material for: The effect of varying thicknesses of mineral trioxide aggregate (MTA) and Biodentine as apical plugs on the fracture resistance of teeth with simulated open apices: a comparative in vitro study
Source: PeerJ. 2024 Dec 18;12:e18691. doi: 10.7717/peerj.18691 (PMC11662899; doi:10.7717/peerj.18691)

**SAMPLE SIZE ESTIMATION**

| **F tests** | ANOVA | Fixed effects, omnibus, one-way |
| --- | --- | --- |
| **Analysis** | A priori | Compute required sample size |
| **Input** | Effect size f | 39.0099157 |
| α err prob |  | 0.05 |
| Power (1-β err prob) |  | 0.80 |
| Number of groups |  | 4 |
|  |  |  |
| **Output** | Noncentrality parameter λ | 12174.19 |
|  | Critical F | 6.5913821 |
|  | Numerator df | 3 |
|  | Denominator df | 4 |
|  | **Total sample size** | 8 |
|  | Actual power | 1.0000000 |


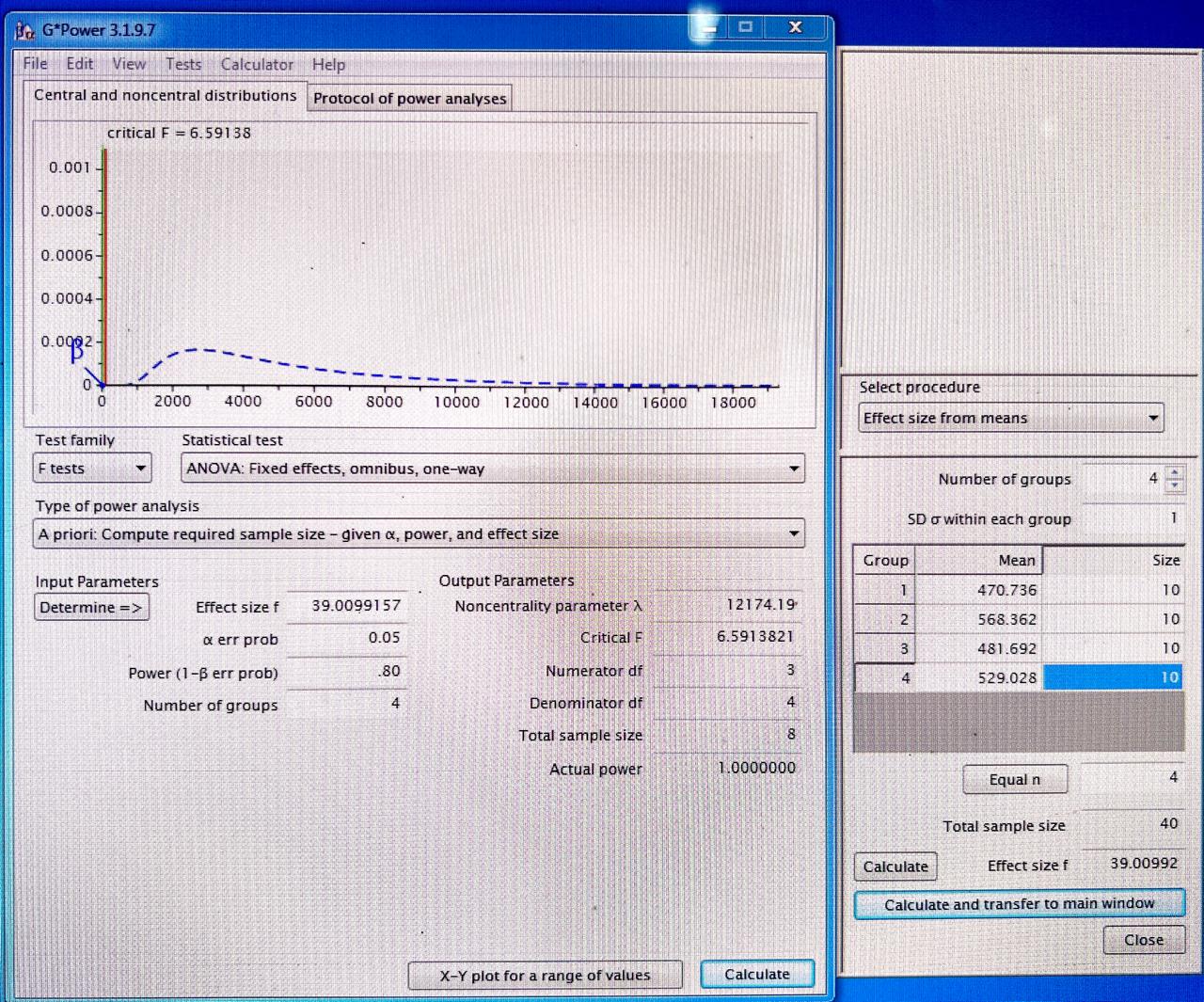

Supplement: Supplemental Information 2 [file peerj-12-18691-s002.docx]
